# Dose and polymorphic genes *xrcc1*, *xrcc3*, *gst *play a role in the risk of articledeveloping erythema in breast cancer patients following single shot partial breast irradiation after conservative surgery

**DOI:** 10.1186/1471-2407-11-291

**Published:** 2011-07-12

**Authors:** Elisabetta Falvo, Lidia Strigari, Gennaro Citro, Carolina Giordano, Stefano Arcangeli, Antonella Soriani, Daniela D'Alessio, Paola Muti, Giovanni Blandino, Isabella Sperduti, Paola Pinnarò

**Affiliations:** 1 Laboratory of Pharmacokinetic/Pharmacogenomic, Regina Elena National Cancer Institute, Rome, Italy; 2 Laboratory of Medical Physics and Expert Systems, Regina Elena National Cancer Institute, Rome, Italy; 3 Department of Radiation Oncology, Regina Elena National Cancer Institute, Rome, Italy; 4 Scientiphic Direction, Regina Elena National Cancer Institute, Roma, Italy; 5 Transational Oncogenomic Unit, Regina Elena National Cancer Institute, Roma, Italy; 6 Scientific Director Office, Regina Elena National Cancer Institute, Roma, Italy

## Abstract

**Background:**

To evaluate the association between polymorphisms involved in DNA repair and oxidative stress genes and mean dose to whole breast on acute skin reactions (erythema) in breast cancer (BC) patients following single shot partial breast irradiation (SSPBI) after breast conservative surgery.

**Materials and Methods:**

Acute toxicity was assessed using vers.3 criteria. single nucleotides polymorphisms(SNPs) in genes: *XRCC1*(Arg399Gln/Arg194Trp), *XRCC3 *(A4541G-5'UTR/Thr241Met), *GSTP1*(Ile105Val), *GSTA1 *and *RAD51*(untranslated region). SNPs were determined in 57 BC patients by the Pyrosequencing analysis. Univariate(ORs and 95% CI) and logistic multivariate analyses (MVA) were performed to correlate polymorphic genes with the risk of developing acute skin reactions to radiotherapy.

**Results:**

After SSPBI on the tumour bed following conservative surgery, grade 1 or 2 acute erythema was observed in 19 pts(33%). Univariate analysis indicated a higher significant risk of developing erythema in patients with polymorphic variant wt *XRCC1*Arg194Trp, mut/het *XRCC3*Thr241Met, wt/het *XRCC3A4541G-5'UTR*. Similarly a higher erythema rate was also found in the presence of mut/het of *XRCC1*Arg194Trp or wt of *GSTA1*. Whereas, a lower erythema rate was observed in patients with mut/het of *XRCC*1Arg194Trp or wt of *XRCC1*Arg399Gln. The mean dose to whole breast(p = 0.002), the presence of either mut/het *XRCC1*Arg194Trp or wt *XRCC3*Thr241Met (p = 0.006) and the presence of either mut/het *XRCC1*Arg194Trp or wt *GSTA1*(p = 0.031) were confirmed as predictors of radiotherapy-induced erythema by MVA.

**Conclusions:**

The Whole breast mean dose together with the presence of some polymorphic genes involved in DNA repair or oxidative stress could explain the erythema observed after SSPBI, but further studies are needed to confirm these results in a larger cohort.

**Trial Registration:**

ClinicalTrials.gov Identifier: NCT01316328

## Background

Breast cancer is the most common type of cancer among women with an incidence rate of 13%. It is also one of the most leading causes of death in the European Union in breast cancer women (88,400 deaths i.e.17.4% of total) [1-3].

Breast radiation therapy (RT) after conservative surgery is now widely accepted as a standard of care for patients with early breast cancer using a multidisciplinary approach. RT destroys the breast cancer cells that remain after surgery, reducing the risk of breast cancer recurrence by about 70%. However RT could also generate radiation-induced side-effects in the surrounding normal tissues [4,5]. An important to address is the unpredictable inter-individual variability in toxicity associated with this treatment [6,7].

Ionising radiation acts directly on cellular macromolecules (DNA, RNA, proteins, etc.), or indirectly through the production of reactive oxygen species and subsequent by-products able to modify bio-molecules. The response of eukaryotic cells to ionising radiation includes and influences several DNA damage repair pathways with subsequent full 'biological' recovery or cell death [8,9]. In particular, *XRCC1 *(x-ray repair cross-complementation group 1) is one of the molecules involved in the restoration phase of the base excision repair (BER) pathway. *XRCC3 *(x-ray repair cross-complementation group 3) allows double strand breaks to be efficiently repaired via the homologous recombination repair pathway, for correct chromosomal segregation and of DNA cross links repair [3,10]. Antioxidant enzymes, such as glutathione-S-transferases (*GSTs*) are activated as a result of RT [11] and are involved in detoxifying of the damage caused by oxidative stress. *RAD51 *is a member of the *RAD51 *protein family, involved in the Homologous Recombination (HR) and repair of DNA. This protein is thought to play a role in homologous pairing and the strand transfer of DNA, and is also found to interact with BRCA1 and BRCA2 in the cellular response to DNA damage.

Various authors [3,8,12-16] have investigated the association between the polymorphic nature of these genes and the possibility of developing biomarkers or predictive assays for radio-sensitivity of tissues acutely treated with ionizing radiation in breast cancer patients. However, further studies are needed to reach any final conclusions.

A number of studies [14,17-21] reported that most (81% - 100%) intra-breast tumour recurrences after breast conserving surgery (BCS) occur in close proximity to the tumour bed, thereby providing the rationale for adjuvant radiotherapy limited to this area [22]. This evidence has supported a new philosophy of Partial Breast Irradiation (PBI). In fact, it has been speculated that by confining the dose delivery to a smaller treatment volume, a larger radiation fraction size could be used without an unacceptable increase in acute/late toxicity. Thus, accelerated partial breast irradiation (APBI) has been promoted in phase I-III trials designed to test the feasibility and equivalence with the standard Whole breast irradiation (WBI) in properly selected low risk early breast cancer patients after BCS.

The aim of this study is to identify marker genes able to predict an increased risk of acute skin toxicity in patients undergoing a single high dose of PBI after BCS at the Regina Elena Italian National Cancer Institute.

In particular, we investigated the following specific polymorphic genes: *XRCC1 *(Arg399Gln, and Arg194Trp), *XRCC3 *(A4541G-5'UTR and Thr241Met), *GSTP1 *(Ile105Val), *GSTA1 *and *RAD51 *(untranslated region) in order to assess the relationship with an increased susceptibility of acute adverse effects induced by radiotherapy.

## Methods

### Radiation treatment

From March 2006 to January 2008, patients who underwent BCS and sentinel node biopsy and/or axillary dissection for early breast adenocarcinoma and who met the eligibility criteria were treated in prone position. An adjuvant single dose 3D conformal radiation therapy (3D-CRT) APBI schedule was administered to the Index Area. The eligibility criteria included: Age ≥ 48 years with a life expectancy of at least 5 years, post-menopausal status, histologically proven, non lobular, adenocarcinoma of the breast, primary tumours ≤ 3 cm, negative surgical margins (≥ 2 mm), negative sentinel nodes or < 4 positive axillary nodes, no extra-capsular extension, no previous radiotherapy. The exclusion criteria comprised of the following: multicentric disease, extended intraductal component (EIC > 25%), Paget's disease, lobular adenocarcinoma, distant metastases. All the above criteria enable the identification of patients at low risk of local recurrence. The study was conducted in accordance with the Helsinki Declaration. Each patient was informed in advance about the study protocol in written form (informed consent) and verbal. The patient was given ample opportunity to request relevant information regarding the study and decide with full autonomy whether to participate in the protocol. The protocol was approved by the local Ethics and Scientific Committee of the Regina Elena Italian National Cancer Institute (reference number IFO-84/10).

From March 2006 to January 2008, 57 patients matched the eligibility criteria and provided a written informed consent. Instead of given an equivalent conventional treatment consisting of 50 Gy (25 daily fractions of 2 Gy in 5-6 week treatments) a single dose was used. The dose of 18 Gy was used in the first 4 patients to test the feasibility of the protocol and whether it was well tolerateda dose a of 21 Gy was administered to the other patients. The dose was delivered to a portion of breast parenchyma corresponding to the tumour bed after surgery. Dose volume constraints were used in order to reduce the dose to normal breast tissue and skin. The major technical details of our approach were reported previously in a separate paper [23].

### Study design

The SSPBI trial was designed as a prospective Phase II single-arm study. The use of a single dose tumour bed is expected to be very effective in terms of tumour control, but it could increase the incidence of radiation induced erythema. Therefore, we assumed that a decreased DNA repair capability, as well as a reduced detoxification of the damage caused by oxidative stress could explain the increased acute toxicity, i.e. a higher incidence of erythema after a single dose. It is for this reason we decided to investigate SNPs of genes involved in antioxidant and DNA damage repair pathways such as *GST*, *XRCC1*, *XRCC3 *and *RAD51*.

We assumed an erythema rate of 20% and 54% in patient groups at low and high risk, respectively, (groups were identified based on the absence/presence of the above polymorphisms alone or in combination). Thus the minimum sample size was 56 patients with α = 0.05, 2-tailed test and a power of the study of 80%.

### End-points

Acute toxicity (i.e. erythema), was the end-point described in the analysis and was assessed using the Regina Elena Italian National Cancer Institute common terminology criteria for adverse events (CTCAE, version 3.0) [24], and was defined as acute if it occurred within the first six months after radiotherapy. Grade refers to the severity of the Adverse Event (AE). Briefly, Grade 1 indicates faint erythema or dry desquamation, G2 moderate to brisk erythema, G3 moist desquamation other than skin folds and creases and G4 skin necrosis of ulceration of full thickness dermis.

### Sample collection and laboratory analysis

All subjects enrolled in the study provided samples of blood, approximately 5 ml, in sterile tubes containing ethylenediaminetetracetic acid (EDTA). Whole blood samples for DNA analyses were immediately frozen at -80°C until processing.

### Molecular analysis

Genomic DNA was isolated from cells in the venous blood using QIAmp kit (QIAmp DNA blood Mini Kit, Qiagen, Valencia, CA), following the manufacturer's instructions, and the DNA quality was evaluated by the spectrophotometer analysis (NanoDrop instrument). Specific primers included in the Radiotherapy Response kit (Diathec Company, Italy) were used in the DNA amplification experiments. The desired DNA was amplified by Real-Time PCR using Rotorgene Instrument (Corbett) following polymerase chain reaction (PCR) conditions provided by the manufacturer. The following polymorphisms were evaluated: *XRCC3 *C18067T (Thr241Met), *XRCC3 *A4541G (5'-UTR untranslated region), *XRCC1 *G28152A (Arg399Gln), *GSTP1 *A313G (Ile105Val) *RAD51 *G135C (untranslated region).

Other polymorphisms such as *GSTA1 *and *XRCC1 *Arg194Trp, which were not included in the above kit, were carried out and tested. The ad-hoc designed primers for these genes are illustrated in Table 1 and PCR reactions were carried out in a volume of 50 μl containing: 10 mM Takara deoxynucleotide triphosphate (dNTP) mixture, 20 pmol primers, approximately 30 ng of DNA template, Takara 5XR-PCR Buffer (Mg^2+^free), Takara 50 mM Mg^2+^, EvaGreen TM Dye 20 × and Takara Ex TaqR-PCR Custom (5U/ul). Reaction conditions were as follows: initial denaturation at 95°C for 3 min then 35-cycles of denaturation at 95°C for 30 sec; annealing at 56°C (*GSTA1) *or at 62°C (*XRCC1 *Arg194Trp) for 30 sec; elongation at 72°C for 30 sec and final extension at 60°C for 5 min, then 5-cycles of green channel signal acquisition at 60°C for 30 sec. PCR products were evaluated on 2.5 % agarose gel stained with ethidium bromide. In addition, one control for each genotype of *GSTA1 *and *XRCC1 *Arg194Trp was generated.

**Table 1:** Polymorphic genes with reference sequence [rs], PCR and sequence primers, products size and annealing conditions

*Gene*	*NCBI* *dbSNP ID*	*Primers*	*PCR* *product* *size (bp)*	*PCR* *Annealing* ***temp***.
*XRCC1 *Arg194Trp	[rs1799782]	*PCR Forward*: 5'-GCCGGGGGCTCTCTTCTT-3' *PCR Reverse biot:* 5'-CTCACTCAGGACCCACGTTGT-3' *Pyrosequence*: 5'-GGGGCTCTCTTCTTCA-3'	75bp	62°C

*GSTA1*	[rs3957356]	*PCR Forward*: 5'-GGCTCGACAACTGAATTCCA-3' *PCR Reverse biot:* 5'-TGGCTTTTCCCTAACTTGACTCT-3' *Pyrosequence:* 5'-GACTCTTCTTTCAGTGGG-3'	101 bp	56°C

**Abbreviations**: NCBI = National Center for Biotechnology Information, ID = identification.

The target sequence containing the polymorphic site was amplified using standard PCR conditions in which one of the primers was biotinylated. A single-strand template was generated by removing the non-biotinylated strand on streptavidin coated beads (matrix). The bound DNA template on the affinity matrix was separated by denaturation in NaOH and used for the following synthesis of a short strand (10-15 bases) of DNA adjacent to the SNP site. The specific sequence primers used for *GSTA1 *and *XRCC1 *Arg194Trp were also reported in Table 1. The polymorphisms were analyzed using Pyrosequencing technologies (instrument PyroMark MD-Biotage, Uppsala, Sweden) according to a previously published method [25,26].

### Statistical analysis

In order to determine predictors of acute toxicity, retrospectively, we evaluated the following polymorphisms: *XRCC3 *C18067T (Thr241Met), *XRCC3 *A4541G (5'-UTR), *XRCC1 *G28152A (Arg399Gln), *GSTP1 *A313G (Ile105Val) *RAD51*, G135C (untranslated region) *GSTA1 *and *XRCC1 *Arg194Trp. In addition, we also analyzed combined genotypes according to the literature.

Odds ratios (ORs) and 95% confidence intervals (CIs), Chi-squared and Fisher exact (2-sided) tests were calculated. A OR > 1.0 indicates an increased risk of erythema in patients with a polymorphic gene.

Patient characteristics, polymorphisms and dosimetric data, selected as significant by the Univariate analysis, were included in a logistic regression analysis in the multivariate model, which included mean dose to whole breast (WB) and to skin, age at the time of treatment and all polymorphisms.

Statistically significant associations between polymorphisms and side effects were indicated by a p-value < 0.05. The sample size was calculated with Sample Power, the odds risks with an R-package, and the Forest plots with Medcalc.

## Results

For the purpose of this study, 57 eligible patients were available for determining polymorphisms. Out of the 57 patients, 15 (26%) were also treated with adjuvant non-concomitant chemotherapy, as reported in Table 1. The adjuvant chemotherapy had been completed 3 to 4 weeks before RT with the exception of one patient (who underwent chemotherapy one-week after SSPBI). Adjuvant hormone-therapy, with Tamoxifen (6 pts), Anastrozole (38 pts) or Letrozole (8 pts), as indicated, were given simultaneously with SSPBI. The median follow-up of the patient groups which underwent SSPBI was 38 months (range 19-50).

However, there was no evidence of chemotherapy induced toxicity at the start of RT. Patient, tumour and treatment related characteristics are listed in Table 2.

**Table 2:** Main patient and tumor characteristics

*Age (years)*	*median (range)*	*66 (51-87)*
**Histology**	*Ductal/other *	48/9
**Estrogen receptor**	*RE+/RE-*	52/5
**Progesteron receptor**	*RP+/RP-*	45/12
**Tumor stage**	*Tis/T1/T2*	1/48/8
**Nodal stage**	*N0/N1*	54/3
**Chemotherapy**	*yes/no *	15/42
	*CMF*	5
	*FEC*	5
	*EC *	1
	*EC+ *Docetaxel	4
**Hormone-therapy**	*yes/no *	52/5

**Abbreviations**: **CMF**: Cyclophosphamide (600 mg/m2, Methotrexate 40 mg/m2, 5-FU 600 mg/m2 d 1 and d 8 q 4 weeks × 6); **FEC **(5-FU 600 mg/m2, Epirubicin 60 mg/m2, Cyclophosphamide 600 mg/m2 d 1 q 3 weeks × 6); **EC **(Epirubicin 60 mg/m2, Cyclophosphamide 600 mg/m2 d1 q 3 weeks × 4); **EC+ Docetaxel **: EC followed by Docetaxel (100 mg/m2 d1 q 3 weeks × 4)

### Polymorphisms

Pyrosequencing is a sequencing method performed by synthesis, a simple to use technique for accurate and consistent analysis of large numbers of short to medium length DNA sequences. Each result, called a "pyrogram", indicates a determined polymorphic position, providing useful information to identify a category of patients with increased risk of toxicity.

**Figure 1:**
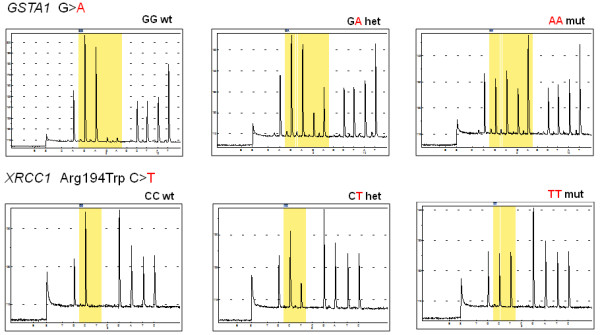
**Typical raw data obtained using Pyrosequencing instruments for *GSTA1 *and *XRCC1 *Arg194Trp polymorphisms**. Note**: **the yellow area indicates the resulting genotypes of *GSTA1 *and *XRCC1 *Arg194Trp.

For *GSTA1 *and *XRCC1 *Arg194Trp polymorphisms that are not included in the Radiotherapy kit, a method assay to validate them was developed and the typical pyrograms obtained are depicted in Figure 1. In this figure, the peak is proportional to the amount of nucleotides incorporated. The sequencing events are obtained in real time and the resulting genotypes of *GSTA1 *and *XRCC1 *Arg194Trp are depicted inside the yellow area. Each gene can generate three distinct pyrosequencing patterns, corresponding to homozygote wild type, heterozygote or homozygote mutant. The polymorphism was determined and the results are shown in Table 3.

**Table 3:** Polymorphism distribution

*Gene*	*SNPs*	*ds SNP ID*	*Observed Genotype* *(n. Pts)*			*allele frequencies(%)*		*%CEU*	
			
			AA	Aa	aa	allele A	allele a	allele A	allele a
*XRCC3*	A4541G	(rs1799794)	32	23	2	78%	22%	74%	26%
*XRCC3*	Thr241Met	(rs861539)	18	33	6	61%	39%	61%	39%
*XRCC1*	Arg399Gln	(rs25487)	31	23	3	75%	25%	57%	43%
*GSTP1*	Ile105Val	(rs1695)	32	22	3	75%	24%	65%	35%
*RAD51*	G135C	(rs1801320)	54	3	/	96%	4%	97%	3%
*GSTA1*	G > A	(rs3957356)	12	35	10	53%	47%	61%	32%
*XRCC1*	Arg194Trp	(rs1799782)	44	12	1	88%	12%	76%	24%

Abbreviations: %CEU = frequencies in European population.

### Acute toxicity

Acute erythema was observed in 19 patients (33%), divided in Grade 1 (G1) in 11 (19%) and Grade 2 (G2) in 8 (14%) patients. No erythema was detected in 38 patients (67%). A statistically significant correlation was found between the ≥G1 erythema and the mean dose to WB (p = 0.008).

The ORs of any grade of erythema (with 95% CI and p-values) are reported in Table 4, by distinguishing the patients on the basis of the polymorphic genes. We found a significant incidence of erythema rate in patients with the polymorphic variant (AA i.e. wt) of *XRCC1 *Arg194Trp with greater odds (OR = 8.07; 95% CI, 1.02-373.8). The allelic variant wt of *XRCC3 *Thr241Met is associated with a lower rate of erythema (OR = 0.29; 95% CI, 0.05-1.29). A toxic role of allelic variant (wt/het i.e AA/Aa) of *XRCC3*A4541G-5'UTR was also found (OR = 0.49; 95% CI, 0.01-40.29).

**Table 4:** ORs of erythema for different polymorphisms and their combination

*Polymorphisms*	*Genotype*	*Percentage* *of ≥G1*	*OR* *(95% CI)*	*Chi-squared p-value*	*Fisher's exact test p-value*
***XRCC1 *Arg194Trp **	(Aa/aa)	8%	1		
	(AA)	41%	8.07 (1.02-373.8)	0.026	0.042
***XRCC3 *Thr241Met**	(Aa/aa)	41%	1		
	(AA)	17%	0.29 (0.05-1.29)	0.07	0.081
***XRCC3 *A4541G (5'UTR)**	(aa)	50%	1		
	(AA/Aa)	33%	0.49 (0.01-40.29)	0.611	1
***XRCC1*Arg194Trp/*GSTP1 ***	Others	50%	1		
	*XRCC1*(Aa/aa) or *GSTP1 *(Aa/aa)	23%	0.3 (0.08-1.08)	0.034	0.046
***XRCC1*Arg194Trp/*XRCC3 *Thr241Met §**	Others	48%	1		
	*XRCC1 *(Aa/aa) or *XRCC3 *(AA)	15%	0.2 (0.04-0.78)	0.008	0.011
***XRCC1 *Arg194Trp/*XRCC1 *Arg399Gln **	Others	46%	1		
	*XRCC1 *Arg194Trp (Aa/aa) or *XRCC1* Arg399Gln (AA)	24%	0.39 (0.1-1.35)	0.088	0.099
***XRCC1 *Arg194Trp/*GSTA1 *#**	Others	11%	1		
	*XRCC1 *(Aa/aa) or *GSTA1 *(AA)	44%	6.01 (1.16-61.04)	0.016	0.018

Abbreviations: OR = odds ratio; calculated to homozygous normal genotype; 95% CI = 95% confidence interval.

§ α = 0.05, 2-tailed power (1-β = 78%; α = 0.05, 1- tailed power > 80%

# α = 0.05, 2-tailed power (1-β = 75%; α = 0.05, 1- tailed power > 80%

Our results show a level that is not statistically significant in the protection towards developing an erythema, i.e. lower odds (OR = 0.3; 95% CI, 0.08-1.08), in the presence of at least one allelic variant (mut/het i.e. aa/Aa) of *XRCC1 *Arg194Trp or of *GSTP1 *(Ile105Val). The presence of the polymorphic variant mut/het of *XRCC1 *Arg194Trp and wt of *XRCC3 *Thr241Met results significantly in the protection towards developing an erythema with lower odds (OR = 0.2; 95% CI: 0.04-0.78).

Furthermore, the association between the polymorphic variant mut/het of *XRCC1 *Arg194Trp and the wt of *XRCC1 *Arg399Gln shows a protective role towards erythema (OR = 0.39; 95% CI: 0.1-1.35). While, the association between the polymorphic variant mut/het of *XRCC1 *Arg194Trp or wt of *GSTA1 *results in a higher risk of erythema (OR = 6.01; 95% CI, 1.16-61.04).

**Figure 2:**
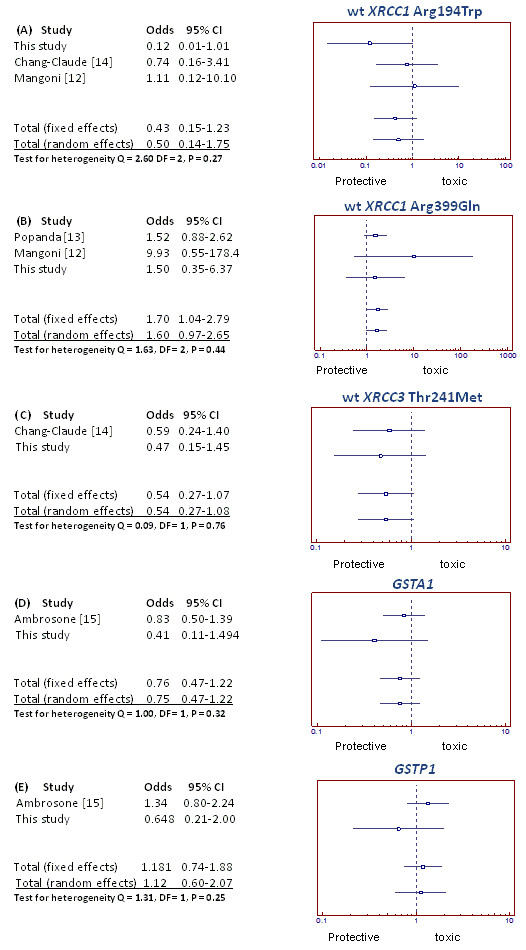
**(a) Forest plot summarizes a pooled analysis of acute erythema distinguishing patients with/without (A) wt *XRCC1 *Arg194Trp; (B) wt *XRCC3 *Thr241Met; (C) wt *XRCC1 *Arg399Gln; (D) *GSTA1*; (E) *GSTP1***. The mutation is toxic or protective when OR is higher or lower than 1, respectively.

Forest plots (Figure 2 panels A-E) show the acute skin toxicity observed in our cohort and in literature, by distinguishing the patients based on the presence of alleles of some polymorphic genes. A protective role (as trend) of mut/het *XRCC1 *Arg194Trp was found, in contrast to data by Mangoni et al. [12] that reported a slightly toxic effect (Figure 2A). A significant function of wt *XRCC1 *Arg399Gln as toxic agent was observed according to data reported by Popanda et al. [13] and Mangoni et al. [12] (Figure 2B). Next, the trend as a toxic factor of the wt *XRCC3 *Thr241Met was found, according to Chang-Claude et al. [14]; as well as of wt *GSTA1*, as reported by Ambrosone et al. [15] who described this gene as protective agent (Figure 2C-D). Further, we found a protective role as trend for wt *GSTP1 *in contrast to Ambrosone et al. [15] (Figure 2E). In fact, no statistical significance for wt *GSTP1 *was found to distinguish acute toxicity in patients in either of the studies. Finally, no correlation was found between acute toxicity and mut/het *RAD51*. Multivariate analysis confirmed the mean dose to WB (p = 0.002), the presence of mut/het *XRCC1 *Arg194Trp or wt *XRCC3 *Thr241Met (p = 0.006) and the presence of mut/het *XRCC1 *Arg194Trp or wt *GSTA1 *(p = 0.031) as predictors of erythema. In addition, when identifying the low and high risk groups, according to the absence or presence of mut/het *XRCC1 *Arg194Trp or wt *XRCC3 *Thr241Met, the observed erythema in our cohort was 20% and 59%, respectively. Assuming an α = 0.05 and a 2-tailed test, the power of the study was 78% with 38 and 19 patients in low and high risk groups respectively; when assuming an α = 0.05 and a 1-tailed test was used the power of the study was 80%.

## Discussion

In this paper we evaluated the effects of polymorphisms in encoding genes for enzymes involved in DNA repair and in protection from ROS (reactive oxygen species), in relation to acute skin side effects of RT. In particular, the following polymorphic genes are involved in oxidative stress-related mechanisms (GSTs) and in DNA repair were taken into consideration: *XRCC1*(Arg399Gln/Arg194Trp), *XRCC3*(A4541G-5'UTR /Thr241Met), *GSTP1*(Ile105Val), *GSTA1 *and *RAD51*(untranslated region), which are involved in oxidative stress-related mechanisms (GSTs) and in DNA repair.

A statistically significant positive connection was found between the ≥G1 erythema and wt *XRCC1 *Arg194Trp, confirming the trend reported by Chang-Claude J et al. [14]. Likewise, an association between *XRCC1 *Arg194Trp and skin reactions was also been described by Mangoni M. et al. [12]. Also in our study, the wt *XRCC3 *Thr241Met showed a trend for having a protective role towards of erythema, according to data reported by Popanda et al. [13] and Mangoni M. et al. [12].

It is worthy to note that, Cecchin et al. [27] investigated the role of *XRCC3*A4541G-5'UTR, a non-coding polymorphism in the 5'UTR of the gene with an un-clarified effect on protein expression, in rectal cancer patients. The Authors suggested a protective role of the variant allele *XRCC3*A4541G-5'UTR toward radiation injury. It is important to stress that a statistically significant positive association correspondence was found between ≥G1 erythema and wt/het *XRCC3*A4541G-5'UTR also in our study, indicating the protective role of the variant allele towards radiation injury also regards to breast cancer. Whereas, no statistically significant correlation was found with other polymorphisms and erythema.

Due to the limited number of studies involving polymorphisms and also the limited number of toxic events reported in literature, a pooled analysis was performed for acute toxicity and was been shown as a Forest plot. This analysis has confirmed the role of some polymorphic variants as predictors of acute toxicity, although in many cases only as a trend (Figure 2).

We also performed tests to assess the association of different genes involved in developmental clinical radio-sensitivity. Particular, we tested whether there was a possible association between the variant mut/het of different genes.

The presence of the polymorphic variant mut/het of *XRCC1 *Arg194Trp or wt of *XRCC1 *Arg399Gln showed evidence of a protective role towards erythema. On the contrary, in the study by Mangoni et al. [12] the presence of *XRCC1 *Arg194Trp variant allele and *XRCC1 *Arg399Gln wt allele suggested a significant risk of toxicity induced by radiotherapy.

In the present study, a correlation was also found between the absence of erythema and the presence of a mut/het of *XRCC1 *Arg194Trp or of the wt *XRCC3 *Thr241Met, indicating the protective role of these alleles.

Further, the association of erythema with the presence of a mut/het of *XRCC1 *Arg194Trp or mut/het of *GSTP1 *was also found to be statistically significant indicating the protective role of these polymorphisms. In addition, a high rate of erythema was correlated with the mut/het of *XRCC1 *Arg194Trp or with of the wt *GSTA1*, while a trend toward having a protective factor was observed when a mut/het of *XRCC1 *Arg194Trp or the wt *XRCC1 *Arg399Gln alleles were expressed.

## Conclusions

This study shows a statistically significant positive relationship between ≥G1 erythema and the wt/het *XRCC3*A4541G-5'UTR polymorphism, indicating a protective role of the variant allele towards radiation injury in BC patients who underwent SSPBI. The reported multivariate analysis suggests that SNP (*XRCC1 *or wt *XRCC3 *Thr241Met and *XRCC1 *or *GSTA1*) and dosimetric data (mean dose to whole breast) may be successfully used to predict acute toxicity in BC patients undergoing RT with/without adjuvant chemotherapy. All of these promising data strongly point to the need for further investigation in order to set up valuable strategies to prevent RT-induced damage in cancer patients.

## Abbreviations

AE: Adverse Event; APBI: accelerated partial breast irradiation; BER: base excision repair; BC: breast cancer; BCS: breast conserving surgery; CEU: frequencies in European population; CIs: confidence intervals; CMF: cyclophosphamide methotrexate 5-fluorouracile; CTCAE: common terminology criteria for adverse events; dNTP: deoxynucleotide triphosphate; EC: epirubicin cyclophosphamide; EDTA: ethylenediaminetetracetic acid; EIC: extended intraductal component; FEC: 5-fluorouracile epirubicin cyclophosphamide; GSTs: glutathione-S-transferases; HR: homologous recombination; ID: identification; MVA: multivariate analysis; NCBI:National Center for Biotechnology Information; ORs: odds ratios; PCR: polymerase chain reaction; PBI: partial breast irradiation; pts: patients; RT: radiation therapy; rs: reference sequence; ROS: reactive oxygen species; SSPBI: shot partial breast irradiation; SNPs: single nucleotides polymorphisms; XRCC1: x-ray repair cross-complementation group 1; XRCC3: x-ray repair cross-complementation group 3; WBI: whole breast irradiation; 3D-CRT: 3D conformal radiation therapy.

## Competing interests

The authors declare that they have no competing interests.

## Non-financial competing interests

The authors declare that they have no competing interests.

## Authors' contributions

EF, PP, LS conceived the study and obtained grant funding, participated in the coordination of the original study, coordinated genotyping efforts, supervised data analysis, and drafted the manuscript. IS, AS and DDA participated in the data management and statistical analysis, and in drafting the manuscript. CG and SA participated in the design of the original study, data collection and patient management, and in drafting the final manuscript. CG, PM, and GB participated in the design of the original study, and participated in drafting the final manuscript. All authors read and approved the final manuscript.

## Pre-publication history

The pre-publication history for this paper can be accessed here:

http://www.biomedcentral.com/1471-2407/11/291/prepub
